# New Insights into Oxidative and Reductive Stress Responses and Their Relation to the Anticancer Activity of Selenium-Containing Compounds as Hydrogen Selenide Donors

**DOI:** 10.3390/biology12060875

**Published:** 2023-06-17

**Authors:** Agnieszka Krakowiak, Sylwia Pietrasik

**Affiliations:** Department of Bioorganic Chemistry, Centre of Molecular and Macromolecular Studies Polish Academy of Sciences, Sienkiewicza 112, 90-363 Lodz, Poland; sylwia.pietrasik09@gmail.com

**Keywords:** reductive stress, oxidative stress, selenium, hydrogen selenide, cancer, H_2_Se donor

## Abstract

**Simple Summary:**

Both oxidative and reductive stress can be destructive to cells. Unlike oxidative stress, reductive stress and the therapeutic opportunities underlying the mechanisms of reductive stress in cancer, as well as how cancer cells reply to reductive stress, have attracted little interest and are not properly characterized. Selenium compounds have been demonstrated to have chemotherapeutic effects against cancer, and their anticancer mechanism is thought to be related to the formation of their metabolites. One of them is hydrogen selenide (H_2_Se), a highly reactive molecule with reducing properties. Unfortunately, most chemotherapeutic selenium compounds were studied under aerobic conditions, although solid tumors exhibit hypoxic conditions. Here, we show the latest reports on the mechanism of cellular recognition and reaction to reductive and oxidative stress as well as the mechanisms through which different types of selenium compounds can release H_2_Se and thereby can selectively affect reductive stress under controlled conditions, which may be crucial for their anticancer activity.

**Abstract:**

Redox balance is important for the homeostasis of normal cells, but also for the proliferation, progression, and survival of cancer cells. Both oxidative and reductive stress can be harmful to cells. In contrast to oxidative stress, reductive stress and the therapeutic opportunities underlying the mechanisms of reductive stress in cancer, as well as how cancer cells respond to reductive stress, have received little attention and are not as well characterized. Therefore, there is recent interest in understanding how selective induction of reductive stress may influence therapeutic treatment and disease progression in cancer. There is also the question of how cancer cells respond to reductive stress. Selenium compounds have been shown to have chemotherapeutic effects against cancer, and their anticancer mechanism is thought to be related to the formation of their metabolites, including hydrogen selenide (H_2_Se), which is a highly reactive and reducing molecule. Here, we highlight recent reports on the molecular mechanism of how cells recognize and respond to oxidative and reductive stress (1) and the mechanisms through which different types of selenium compounds can generate H_2_Se (2) and thus selectively affect reductive stress under controlled conditions, which may be important for their anticancer effects.

## 1. Introduction

Redox homeostasis, that is, the proper balance between pro- and antioxidants, is important for almost all natural processes [1]. The functioning of mammalian cells depends on several oxidation-reduction reactions to generate energy (e.g., synthesis of ATP) and to produce important cellular factors (such as nucleic acids) from nutrients to assist their biological functions. These redox reactions are critical for the homeostasis of normal cells as well as for the proliferation, development, and survival of tumor cells [2]. Under physiological conditions, when cells are exposed to reductive or oxidative insults, cellular redox buffers have sufficient capacity to maintain cellular oxidants and reductants at physiological levels. The balance between molecular redox pairs, such as GSSG/GSH, NAD(P)^+^/NAD(P)H, and FAD/FADH_2_ (glutathione disulfide/reduced glutathione, nicotinamide adenine dinucleotide(phosphate)/reduced nicotinamide adenine dinucleotide(phosphate), and flavin adenine dinucleotide/reduced flavin adenine dinucleotide) is essential for the regulation of various signaling processes responsible for maintaining intracellular homeostasis, and, collectively, these ratios design the cellular redox state [3]. These redox pairs function as cofactors or substrates for the enzymatic or non-enzymatic neutralization of electrons and reactive oxygen species (ROS), including maintenance of a relatively reducing environment in cells and participation in cellular energetics. For instance, NADPH is an important electron source for the biosynthesis of fatty acids and nucleic acids, NADH delivers electrons for oxidative phosphorylation (oxPHOS) in mitochondria, and NAD^+^ serves as an electron scavenger to support glycolysis [4].

When the equilibrium between cellular oxidation and reduction potential is shifted in favor of the oxidizing fraction, a phenomenon called oxidative stress is observed. It occurs when the cell’s ability to defend itself against antioxidants is overwhelmed by the massive production of pro-oxidants such as ROS/RNS/RSS (reactive oxygen species/reactive nitrogen species/reactive sulfur species) [5,6]. Redox imbalance towards pro-oxidative conditions is involved in cancer development as oxidative stress causes genomic instabilities, favoring cancer metastasis and progression [2].

On the other hand, if the cellular redox balance is shifted towards reduction, reductive stress is observed, defined as excessive accumulation or production of reducing agents, such as GSH, NADH, NADPH, as well as the free thiol groups in proteins present in cysteine residues. These cysteines in reduced form, which are present in excess in proteins, can lead to the activation of the “Unfolded Protein Response” (UPR) [7], which impairs the activity of endogenous oxidoreductases. In addition, reductive stress lowers cellular ROS levels below their physiological levels, disrupting their signaling functions. From another perspective, reductive stress may also promote the production of ROS, as redox couples can reduce O_2_ to ^●^O_2_^−^ in an oxygen environment [3,8,9].

Imbalance of redox status may result from contact with infectious factors or certain diseases. Oxidative stress plays a role in the development of many human diseases, including cancer, as well as aging. Different levels of redox balance affect the regulation of cellular processes in tumors in different ways [10]. Cancer cells generally exhibit a more oxidized environment, meaning that their redox balance is shifted toward higher levels of ROS, which plays a critical role in tumor development, e.g., initiation, progression, migration, invasion, and metastasis. Moderate concentration of ROS promotes the proliferation and metastasis of cancer cells, favoring tumor progression. This is because higher levels of ROS may be the result of more intense oxidative phosphorylation in mitochondria, which means higher ATP production, since cancer cells require a lot of energy for growth. However, oxidative stress, when enormously high, is toxic through oxidative damage to intracellular biomacromolecules in cancer cells [10]. Depending on the stage of cancer development, cells are able to adapt to high ROS levels via altering their metabolism through various mechanisms. These include the activation of antioxidant transcription factors, the elevation of NADPH via the pentose phosphate pathway (PPP), and reductive glutamine and folate metabolism, all of which allow cancer cells to survive [11]. Several antioxidant enzymes and molecules are overexpressed under oxidative stress conditions in cancer and often this metabolic reprogramming leads to a state of ‘pseudohypoxia’ [12]. 

On the other hand, reductive stress impairs cellular signaling and function and has been associated with cancer, diabetes, and cardiomyopathy [13]. Reductive stress can lead to disruption of mitochondrial homeostasis, decrease metabolism, influence resistance to anti-cancer therapies, alter the formation of disulfide bonds in proteins leading to activation of UPR/ER stress [3], and finally be harmful to cells. Redox biology, therefore, seeks to understand the mechanisms of regulation and maintenance of homeostasis, as well as the processes that are perturbed in various disease progress where oxidative or reductive stress is a problem [14].

Chemotherapeutic agents are designed to kill cancer cells because, as antioxidants, they usually act through shifting the redox balance toward reductive stress, which paradoxically can force cells to produce an excess of ROS and, consequently, generate oxidative stress [3,9,15]. This mechanism resembles an uncontrolled amplification of cellular antioxidant signaling leading to reductive stress [10]. However, under hypoxic conditions, which are a feature of the microenvironment of solid tumors, only a small amount of O_2_ is present, limiting the production of ROS [16]. Therefore, it is of great interest to understand how selective induction of reductive stress may influence therapeutic treatment and disease progression in cancer, particularly under conditions of limited O_2_ levels. Consequently, it is important to recognize that the effects of chemotherapeutic agents may differ under different oxygen conditions. Over the years, many selenium-containing compounds have been investigated as anticancer chemotherapeutic agents, but the specific mechanism of their anticancer activity has not been fully elucidated [17,18,19,20]. It has been suggested that their metabolites, such as methylselenol (CH_3_SeH) and/or hydrogen selenide (H_2_Se), may be responsible for their anticancer effects. What is important, cancer cells have been found to be significantly more sensitive than normal cells to the antiproliferative effects of many selenium-containing compounds [17]. Hydrogen selenide is a highly reactive and reducing molecule and thus can induce a reductive environment in cells. Therefore, selenium compounds that are H_2_Se donors may selectively induce reductive stress and be useful in anticancer and redox homeostasis research. However, the mechanism of H_2_Se formation from various selenium compounds may vary in cells, and there are not many compounds whose H_2_Se release is well documented, as we will discuss in this review. 

Unlike oxidative stress, reductive stress is a phenomenon that is not sufficiently described. Little attention has been paid to reductive stress and the therapeutic possibilities underlying the mechanisms of reductive stress in cancer, as well as how cancer cells respond to reductive stress, and there are many conflicting hypotheses in the literature [2,9]. This concept was first described by Gores et al. in 1989 [21]. The authors performed experiments in which they induced hypoxia using chemicals and blocked mitochondrial respiration and ATP production in rat hepatocytes. They concluded that inhibition of respiration leads to “reductive stress”, which can contribute to lethal cellular damage due to low oxygen levels and the formation of toxic oxygen species. These findings called for further studies on this new concept and the mechanism of reductive stress.

On the other hand, the term oxidative stress was first used in 1970 by Paniker et al. during studies on GSH/GSSG pairs in H_2_O_2_-stimulated normal and GR-deficient human erythrocytes [22]. Since then, the number of reports of oxidative stress in the literature has increased significantly, from 14 in 1980 and 242 in 1990 to 12,356 in 2010 and 29,069 in 2022 (based on PubMed). 

Metabolic responses to reductive stress such as biosynthesis and cellular distribution of GSH, metabolic sources, and cellular distribution of NAD(H)^+^ and NADP(H) couples were reviewed in 2020 [3]. The role of redox homeostasis in the growth, survival, and resistance to therapy of cancer cells has also been recently presented [10]. Here, we present recent reports on how cells detect and respond to oxidative and reductive stress at the molecular level. We also highlight the mechanisms of H_2_Se formation from various types of selenium compounds. These compounds have the potential to selectively induce reductive stress and potentially may act as chemotherapeutic agents in cancer.

## 2. Redox Network in Mammalian Cells

Optimal levels of ROS are critical for intracellular signal transduction for proper cellular functions. Redox signaling may play a crucial role in immune response, stem cell biology, cancer, and aging [23]. Imbalance of redox status is observed in the development of many human diseases such as obesity, diabetes, neurodegenerative diseases, and cancer. This imbalance may result from exposure to infectious factors (e.g., viruses and bacteria) but also from exposure to radiation and toxins [10]. In tumors, redox imbalance and the subsequent disruption of redox signaling are associated with the proliferation and development of cancer cells and their resistance to radio- and chemotherapy. 

Organisms have preserved stress response pathways that recognize and mitigate a wide range of adverse conditions to protecting cell populations from harm [23]. Due to their quick activation, stress responses must be turned off shortly after cellular homeostasis is reinstated; otherwise, the cell faces far-reaching consequences, including death. The molecular responses to oxidative and reductive stress are shown below, demonstrating the ability of the cellular machinery to respond rapidly.

### 2.1. Detection and Response to Oxidative Stress

Oxidative stress manifests as increased production of ROS/RNS/RSS generated by enzymes such as nicotinamide adenine dinucleotide phosphate oxidases (NADPH oxidase) and nitric oxide synthases (NOSs) or the system of electron transport chain in mitochondria (mtETC) [5,6]. The redox potential of redox pairs as well as the concentration of ROS vary depending on their localization in the subcellular compartments [14]. Thus, H_2_O_2_ concentration was found to be about 80 pM in the cytosol, 20 nM in the mitochondria, 700 nM in the endoplasmic reticulum, and 1–5 μM in the extracellular space. To counteract ROS/RNS/RSS accumulation, various antioxidant systems are active (oxidative stress response), including non-enzymatic ones such as GSH, ascorbate, and α-tocopherol (vitamins); enzymatic ones such as superoxide dismutases (SODs), catalase, glutathione reductase (GR), glutathione peroxidases (GPxs), thioredoxins (Trxs), and peroxiredoxins (Prxs); and others, such as nuclear factor erythroid 2-related factor (NRF2) [5,6,24]. NRF2 is the major transcription factor of antioxidant defense that regulates many genes encoding antioxidant response through inducing the expression of proteins that scavenge oxidizing molecules and convert oxidized proteins to their functional reduced state [25,26,27,28]. NRF2 also induces the expression of detoxification enzymes and suppresses the induction of pro-inflammatory cytokine genes [29,30]. Stem cells that are unable to shut down the oxidative stress response are unable to establish the physiological ROS required for signal transduction and differentiation [31,32]. On the other hand, NRF2 function has been shown to be overactivated in many cancers and such aberrant NRF2 activation in cancer cells strongly correlates with negative clinical prognosis [33,34,35].

Stress responses are often controlled via ubiquitination, the specificity of which depends on many different E3 ligases conjugating activated ubiquitin to the substrate [36,37,38]. This posttranslational modification enables mechanistically diverse, quantitative, and reversible regulation of various cellular processes, including signal transduction, migration, cell division, and differentiation. On the other hand, abnormal ubiquitination leads to a broad spectrum of developmental diseases, cancer, and neurodegeneration [39].

**Figure 1:**
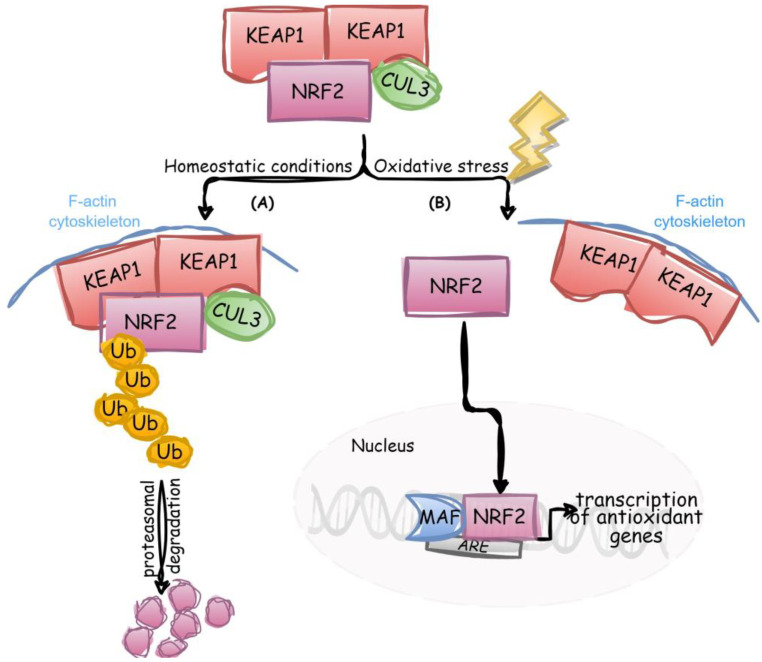
Scheme of NRF2 and KEAP1 interactions [41]. (**A**) Under normal conditions, NRF2 is bound to its cellular inhibitor, KEAP1, which is connected with the F-actin cytoskeleton and is inactive. NRF2 concentrations are mostly regulated by the proteasome [52,53]. (**B**) In the response to oxidative stress, NRF2 detaches from KEAP1, translocates to the nucleus and forms a heterodimer with MAF: the NRF2/MAF heterodimer binds to *ARE* and prompts the expression of different genes, including antioxidant. (Adapted from ref. [43]; reproduced with permission of Elsevier).

In oxidative stress, stabilization of NRF2 transcription factor activity is critical. Under normal conditions, in the absence of activation signals and when high levels of this transcription factor are not required, NRF2 interacts with KEAP1 (Kelch-like erythroid cell-derived protein with CNC homology-associated protein 1) [40], which is associated with the F-actin cytoskeleton and has been described as an important sensor of oxidative stress in the cell [25,41]. The stoichiometry of KEAP1 and NRF2 within the complex is 2:1, as demonstrated in an isothermal calorimetry study [42]. Then, NRF2 is ubiquitinated with the participation of E3 ligase cullin-3 (CUL3) and subsequently degraded via the proteasome (Figure 1A). When cells are exposed to oxidative stress, the formation of the NRF2/KEAP1/CUL3 complex is inhibited by ROS-dependent oxidation of Cys residues in KEAP1. Several reports have demonstrated that Cys151/273/288/226/613/622/624 may be responsible for the multiple mechanisms of stress-sensing acting through KEAP1 [40,43,44,45]. Consequently, the sequestration and ubiquitination of NRF2 are stopped, and NRF2 translocates and accumulates in the nucleus, where it heterodimerizes with MAF (musculoaponeurotic fibrosarcoma oncogene homolog) protein. The heterodimers recognize the *ARE*s, which are enhancer sequences in the regulatory regions of NRF2 target genes, essential for the transcription and expression of antioxidant genes [41,46] (Figure 1B).

Similarly, the E3 CUL2/VHL complex constrains HIF-1α (hypoxia-inducible factor 1) until hypoxic stress occurs, and this released transcription factor then initiates angiogenesis [47]. HIF-1α is a key transcriptional regulator of cellular metabolism under hypoxic conditions, which also occur in solid tumors. This transcription factor is involved in regulating the expression of many genes responsible for the differences in metabolism compared with the corresponding normal tissue, and there is evidence that (pseudo)hypoxia increases cellular GSH levels via both HIF-1α-dependent and -independent mechanisms [48].

The importance of correct stress signaling can be illustrated by the deletion of *VHL* or *KEAP1*, which leads to embryonic or early postnatal death, respectively [49,50], and their mutations are a common cause of cancer, e.g., lung squamous cell carcinoma [47,51].

### 2.2. Detection and Response to Reductive Stress

Reductive stress manifests as ROS depletion below their physiological level, due to prolonged antioxidant signaling or mitochondrial inactivity that reverses oxidation. Reductive stress manifests as excessive levels of reduced NAD+ (NADH), reduced NADP+ (NADPH), and GSH. It is as damaging as oxidative stress and has been associated with many pathological processes [3,10]. For example, elevated GSH levels lead to increased resistance to chemotherapeutic agents in numerous cancers, as some cancer cells can develop drug resistance through redox resetting [54], which can promote cancer migration and metastasis. Similar to ROS, the distribution of NAD(H), NADP(H), and GSH/GSSG is highly compartmentalized in the cell. Unexpectedly, the production of ROS can also increase because of reductive stress, i.e., via activation of NOXs, one-electron transfers to oxygen or through ETC complexes I and IV [8].

Reductive stress may be the result of, among other things, excessive activation of NRF2 as a result of an oxidative stress response that is not shut down quickly enough, which may occur in cancer. This can lead to overexpression of, e.g., G6PD (glucose-6-phosphate dehydrogenase), the NADPH-producing enzyme. Therefore, similar to rapid activation, stress responses have to be turned off shortly after cellular homeostasis has been reestablished. Thus, cells which do not shoot down the oxidative stress response are incapable of collecting the physiological ROS levels necessary for signaling and are not able to differentiate because of the consequential reductive stress [23,31,41]. An extended lack of ROS, named reductive stress, also blocks glucose homeostasis and insulin signaling (manifesting as increased expression of an antioxidant enzyme such as GPX1) [55], triggers cardiomyopathy (through dysregulation of G6PD activity) [26], muscular dystrophy (due to cytoplasmic laminin aggregation and activation of the NRF2/KEAP1 pathway) [56], or diabetes (NADH/NAD^+^ redox imbalance due to impairment of NAD^+^ regeneration enzymes) and increases mortality [57]. Some proteins, which are redox-sensitive and are associated with cell growth and survival, such as PTEN (phosphatase and tensin homolog; tumor suppressor), NF-κB (nuclear transcription factor kappa B that is involved in inflammatory and immune responses and in the regulation of expression of many other genes related to cell survival, proliferation, and differentiation), Ref1 (redox factor-1), p53 (transcription factor of genes associated with cell cycle, DNA repair, apoptosis, etc.; tumor suppressor protein), and PPATδ (peroxisome proliferator-activated receptor δ; modulates glucose and lipid metabolism), have also been shown to be involved in the reductive stress response in cancer [10].

**Figure 2:**
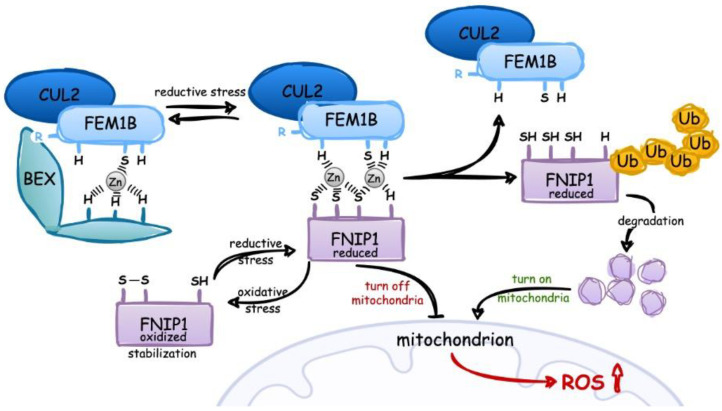
Scheme of reductive stress signaling. Reductive stress reverses oxidation state of thiols in Cys residues in FNIP1, leading to the recognition of FNIP1 by ligase complex CUL2/FEM1B via Zn^2+^-dependent binding, ubiquitination, and degradation in proteasome (modified from [58]). Decreasing concentration of FNIP1 intensifies mitochondrial activity and starts the generation of ROS to neutralize reductive stress. The binding of reduced form FNIP1 to the ligase complex CUL2/FEM1B is controlled by BEX family of proteins (modified from [59]).

Despite these serious consequences, only recently Manford et al. have reported how reductive stress is sensed and alleviated [58]. They found that cells detect and respond to reductive stress through ubiquitinating and degrading the mitochondrial gatekeeper FNIP1 (Folliculin-interacting protein 1). The complex E3 ligase CUL2/FEM1B (Cullin-2 ligase/Fem-1 homolog B) can bind its target (FNIP1) in a redox state-dependent manner. Reductive stress, as caused by prolonged antioxidant signaling or mitochondrial inactivity, reverses the oxidation of Cys residues in FNIP1. This means that the Cys residues are in reduced form, which allows the ligase CUL2/FEM1B to recognize and bind FNIP1 through direct interaction with FEM1B (Figure 2). Based on structural data from X-ray crystallography, these interactions were found to occur via zinc ions at the interface between FEM1B and the cysteine residue C186 in FNIP1, which is critical for the recognition of the FEM1B substrate [59]. As a result, CUL2/FEM1B-dependent FNIP1 ubiquitination and proteasomal degradation of FNIP1 happen, followed by restoration of mitochondrial production to generate ROS and maintain redox homeostasis. It is speculated that regulation of this process can occur with the involvement of BEX proteins (brain-expressed and X-linked proteins), which are pseudosubstrate inhibitors of E3 ligase, and participate in the reductive regulation of the stress response (Figure 2). BEX proteins bind multivalently to the ubiquitination ligase via sites located at C186/Zn^2+^ and R126 of FEM1B. Recently, the small synthetic ligand EN106 was shown to target a cysteine residue in FEM1B that is essential for substrate recognition [60]. Through covalent interactions with C186, EN106 is able to bind FEM1B E3 ligase and disrupt the recognition of its key reductive stress substrate, FNIP1. It may be of future interest to determine whether EN106 can be used therapeutically to inhibit the formation of the CUL2/FEM1B/FNIP1 complex and disrupt reductive stress signaling through stabilizing FNIP1 in certain cancers.

In summary, the key to the reductive stress response is the ability of the E3 ligase CUL2/FEMB1 to distinguish the reduced from the oxidized form of FNIP1, so the E3 ligase is able to discriminate targets based on redox state with zinc ions at the interface (it selectively recruits the reduced Cys residues of FNIP), and this interaction is controlled by BEX family pseudosubstrate inhibitors. Finally, the degradation of FNIP1 leads to the activation of mitochondria to recalibrate ROS (Figure 2).

Thus, the reductive stress response relies on ubiquitin-dependent regulation that turns mitochondrial activity on and off according to redox demand and involves metabolic control in the coordination of stress and developmental signaling.

Recent evidence suggests that reductive stress may be an avenue for therapeutic intervention in cancer [61]. Under hypoxic conditions, CTMP-AA nanocarrier (a core–shell nanostructure of CdTe quantum dots coated with mesoporous silica (MSN), functionalized with poly(2-vinylpyridine)-polyethylene glycol-folic acid (PPF), and loaded with ascorbic acid) controlled release of high levels of ascorbic acid can trigger apoptosis of HepG2 cells (human hepatocellular carcinoma) through inducing reductive stress, with apparently limited damage to normal tissue. In vivo studies in mice bearing HepG2 tumor confirmed that CTMP-AA exhibits a killing effect on tumor cells and inhibits tumor growth.

## 3. The Biological Significance of Selenium Compounds

Selenium is an essential trace element whose role is important in health and disease. It is usually supplied in the daily diet in the form of inorganic (drinking water) or organic (plants, yeasts, and animal sources) compounds such as selenate (sodium selenate), selenite (sodium selenite), selenocysteine (Sec), selenomethionine (SeM), and less commonly methylselenocysteine (MeSeCys) and CysSeSeCys [62]. Many cellular physiological processes depend on the level of selenium compound intake. At relatively low concentrations (about 55 µg/day), Se is necessary for various physiological processes, while at high doses (>400 µg/day), it can have toxic effects and is also harmful to health when deficient (dose below 20 µg/day) [17].

**Figure 3:**
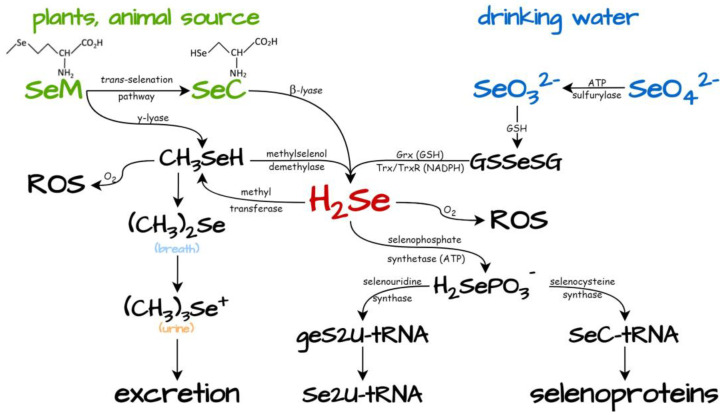
A simplified scheme of endogenous H_2_Se production and metabolism. SeO_4_^2−^, selenate; SeO_3_^2−^, selenite; SeC, selenocysteine; SeM, selenomethionine; Grx, glutaredoxin; GR, glutathione reductase; Trx, thioredoxin; TrxR, thioredoxin reductase; GSH, reduced glutathione (tripeptide γ-Glu-Cys-Gly); GSSeSG, selenodiglutathione; GR, glutathione reductase; S2U, 2-thiouridine; Se2U, 2-selenouridine; ge, geranyl group; geS2U-tRNA, geranyl group at S2U of tRNA.

Dietary Se compounds are metabolized in vivo via different pathways to different selenium metabolites, but their pathways meet in a shared metabolite, recognized as hydrogen selenide (H_2_Se, mainly as HSe^−^ at physiological pH), which is a reduced form of selenium (Figure 3, section “Dietary selenium compounds as generators of H_2_Se”) [20]. H_2_Se is a highly reducible molecule with very high chemical reactivity that cannot be readily detected in cells and animal models. It is efficiently taken up by human cells with an apparent K_m_ in the nanomolar range through transport via ATPases, allowing it to be readily distributed in tissues [63]. 

Selenium compounds mediate physiological functions mainly through the incorporation of Se into selenoproteins, usually in the form of selenocysteine (Sec), the 21st amino acid. For the incorporation of selenocysteine into selenoproteins, selenium has to be converted to selenide, from which monoselenophosphate (H_2_SePO_3_^−^) is formed to generate selenocysteyl-tRNA^(Ser)Sec^ (SeC-tRNA) [64,65]. Finally, only selenium compounds that can be converted to selenide can serve as selenium sources for selenoproteins [62]. Most selenoproteins, such as GPxs and TrxRs, incorporate Sec into their active sites and exhibit antioxidant properties—they protect the cell from the harmful effects of ROS. On the other hand, SeM is randomly integrated into proteins. The antioxidant property of selenium-containing proteins (chemopreventive effect) has been used to prevent cancer, and this result occurs at low levels of dietary Se compounds [20,66]. Conversely, selenium shows an antitumor activity (chemotherapeutic effect) at relatively high levels [17]. Several Se compounds showed significant cytotoxicity (in the low micromolar range) against various malignant cells (lung, prostate, cervical, etc.) [2,17,18]. However, the effects of selenium application are highly dependent on the chemical form, cell type, and biomarker used to test efficacy [62].

The other physiological function of selenium compounds is exerted by selenium modification of tRNA, which has been found so far in bacteria (Figure 3). The enzyme tRNA 2-selenouridine synthase (SelU) is responsible for a two-step process: (1) S2U-RNA (2-thiouridine RNA) geranylation (with ppGe) and (2) subsequent selenation of the resulting geS2U-RNA (with SePO_3_^3−^) to Se2U-RNA (2-selenouridine RNA) [67,68]. Se2U or S2U in the wobble position of the anticodon fragment of some tRNA plays an important role in the precise reading of mRNA during translation through increasing the thermodynamic stability of RNA duplexes containing S2U-A or Se2U-A base pairs and restricting the formation of S2U-G or Se2U-G base pairs [69].

Excretion of excess selenium (detoxification) occurs through sequential methylation of H_2_Se to dimethylselenide ((CH_3_)_2_SeH), which is excreted in the breath, and selenosugar and trimethylselenide ((CH_3_)_3_Se^+^), which are excreted in the urine.

## 4. Selenium Compounds as H_2_Se Donors That May Affect Redox Homeostasis in Healthy Organisms and Cancers

Several selenium compounds have been shown to have chemotherapeutic effects against cancer [17,18,20], but their anti-cancer mechanism has not been fully elucidated. It is suggested that H_2_Se or methylselenol (CH_3_SeH), the common metabolites of several selenium compounds, including some chemically synthesized ones, may be the actual anticancer agents [18,19,70,71,72,73]. Therefore, research on existing and new selenium-containing drugs with antitumor effects should focus on these molecules. Excessive amounts of selenide or methylselenol may be harmful to cells [17,20]. In oxygen environment, these two forms readily oxidize and can lead to the formation of superoxide and other reactive oxygen species with additional toxic effects. The most of the chemotherapeutic selenium compounds have been studied under aerobic conditions, and the generation of oxidative stress has been assumed the main mechanism of their anticancer activity under these conditions [17,20]. However, in solid tumors, that is, under low-oxygen conditions, e.g., hypoxia, high H_2_Se concentrations were produced after administration of an external selenium source, without obvious increase in ROS being observed, and H_2_Se accumulation led to reductive stress in studied cells [74].

Thus, the anticancer effects of various selenium compounds are related to their influence on the redox homeostasis of cancer cells, but the question arises whether Se is an antioxidant or a pro-oxidant. 

The direct role of hydrogen selenide in cancer therapy with selenium compounds had not been investigated until 2016 [75,76] (see Section 4.1), probably due to the lack of well-characterized and controllable H_2_Se donors under physiological conditions, and the lack of detection methods for this highly reactive, reducible, and short-living molecule. The half-life for the oxidation of H_2_Se (HSe^−^ at physiological pH) to elemental selenium in air-saturated water (without other reactants) and at pH 7 is about 30 s, and independent of its concentration [77]. H_2_Se has analogous physical and chemical properties to H_2_S (hydrogen sulfide) but also shows significant differences; therefore, it exerts different biological effects because selenium compounds are mostly more reactive than corresponding sulfur products [78]. Unlike H_2_Se, the half-life time of H_2_S in air-saturated water is a few minutes. Sulfur and selenium belong to the same group in the periodic table of elements and have a similar atom radius (88 and 103 pm), similar covalent radius (102 and 116 pm), and similar electronegativity (2.58 and 2.55). They also show some differences, since a selenium atom is a better-reducing agent and oxidizes more easily, while a sulfur atom tends to form much stronger covalent linkages. The pK_a1_ values of H_2_S and H_2_Se vary significantly (6.88 and 3.89), while the pK_a2_ values of HX^−^ do not differ considerably (14.15 and 15.1, respectively). H_2_S, the gaseous, membrane-permeable molecule, is the central compound of cellular sulfur chemistry and is one of three inorganic gaseous messengers in mammalian cells (the others are NO and CO). The formation of reactive species (RSS) from H_2_S is analogous to ROS so that the transfer of single electrons starting from H_2_S leads to the RSS thiyl radical (HS^●^), hydrogen persulfide (H_2_S_2_), supersulfide radical (^●^S_2_^−^), and finally to elemental sulfur [14]. Due to the higher reactivity of selenium compounds compared to the corresponding sulfur derivatives, it can be argued that all biologically relevant selenium compounds are “Reactive Selenium Species” RSeS) [78]. Therefore, H_2_Se as RSeS should generate oxidative stress similarly to ROS and RSS. On the other hand, H_2_Se, a molecule with strong reductive properties, is theoretically capable of inducing reductive stress through lowering cellular ROS below their physiological levels and disrupting their signaling functions. As a result, reductive stress can promote the production of ROS as redox pairs can reduce O_2_ to form ^●^O_2_ ^−^ leading to DNA strand breaks and apoptosis in cancer cells [3,9]. 

However, under hypoxic conditions, H_2_Se accumulation can lead to reductive stress [74]. Thus, the toxicity of selenium-containing compounds likely combines the dependence on several mechanisms, such as affecting the redox state and eventually DNA damage/repair, gene expression, cell signaling pathways, cell cycle arrest, GSH levels, protein oxidation (in the presence of O_2_), the angiogenesis process, metastasis, or others [17,70]. Additionally, the chemical form in which they are supplied to the organism (e.g., organic or inorganic) is also important and can have an influence on the effect exerted.

### 4.1. Dietary Selenium Compounds as Generators of H_2_Se

Selenomethionine (SeM), selenocysteine (SeC), selenite (SeO_3_^2−^), and selenate (SeO_4_^2−^) account for the majority of organic and inorganic Se in the diet. They are absorbed without regulation, metabolized in a different way in vivo, and they generate different selenium metabolites, but their common intermediate is H_2_Se (Figure 3) [62]. Organic selenium compounds released from selenoproteins (from plant and animal sources) such as SeM and SeC are absorbed via transcellular pathways. SeM can enter the *trans*-selenation pathway (analogous to the *trans*-sulfuration pathway) and be converted to SeC, which is subsequently reduced to H_2_Se (HSe^−^) by selenocysteine β-lyase. Alternatively, SeM can be converted into methylselenol (CH_3_SeH) by a γ-lyase and then to H_2_Se by methylselenol demethylase. 

Sec, SeM, and MeSeCys are seleno-amino acids that have been studied for their anticancer properties. Sec inhibits the growth of cancer cells (lung, breast, bladder, liver, and other) through inducing apoptosis and cell cycle arrest in phase S, increasing DNA fragmentation, and activating the caspase-3 via the mechanism related to the overproduction of ROS [18,79,80]. Inhibition of cell proliferation through apoptosis induction caused by SeMet has been observed in various cancer cell lines, such as colorectal, prostate, breast, lung, and melanoma cells [18,81]. However, MeSeCys is generally more toxic than SeM as shown in lung cancer (A549) and human neuroblastoma cells (SH-SY5Y), probably due to its quick metabolism to CH_3_SeH, a potentially antitumorigenic compound [71,72,82,83]. Therefore, the antitumor effect of these dietary organic selenium compounds is thought to be due to the formation of CH_3_SeH and its further oxidation rather than the involvement of H_2_Se, although this pathway cannot be excluded due to a lack of evidence. 

On the other hand, SeO_3_^2−^ and SeO_4_^2−^, the primary environmental inorganic Se sources, which are mainly supplied with drinking water, are metabolized differently. After absorption, inorganic selenate is reduced to selenite by ATP sulfurylase (Figure 3). SeO_3_^2−^ reacts spontaneously with GSH to form selenodiglutathione (GSSeSG) [78], and subsequently, GSSeSG is decomposed to selenide in the presence of NADPH by the enzymatic cellular system in several steps. The enzymes involved in these redox reactions are GR, glutaredoxin (Grx), or the Trx/TrxR system (selenium proteins themselves) [62,84]. Next, in the presence of oxygen, H_2_Se is easily oxidized by O_2_ to Se^0^. However, excess GSH can protect H_2_Se from oxidation through trapping O_2_ and forming GSSG, so the role of GSH is to protect functional protein groups from oxidation. Therefore, depletion of intracellular GSH by H_2_Se can lead to oxidation and loss/decrease in the function of different proteins necessary for maintaining cell homeostasis.

SeO_3_^2−^ constitutes an inorganic form of selenium that has cytotoxic effects on several human cancer cell lines, including glioma, prostate, and lung [85,86,87,88]. For example, comparative proteomics and mass spectrometry studies demonstrated that sodium selenite induced significant growth inhibition and apoptosis in the human prostate cancer cell line PC-3 [89]. Na_2_SeO_3_ increased intracellular ROS and decreased mitochondrial membrane potential, leading to activation of caspase-8, cleavage of PARP, and finally apoptosis of cells. Selenite-induced apoptosis was mediated mainly by the mitochondrial pathway but also involved ER stress and HIF-1α mediated pathways. Sodium selenite also inhibited the migration of PC-3 cells in vitro.

**Figure 4:**
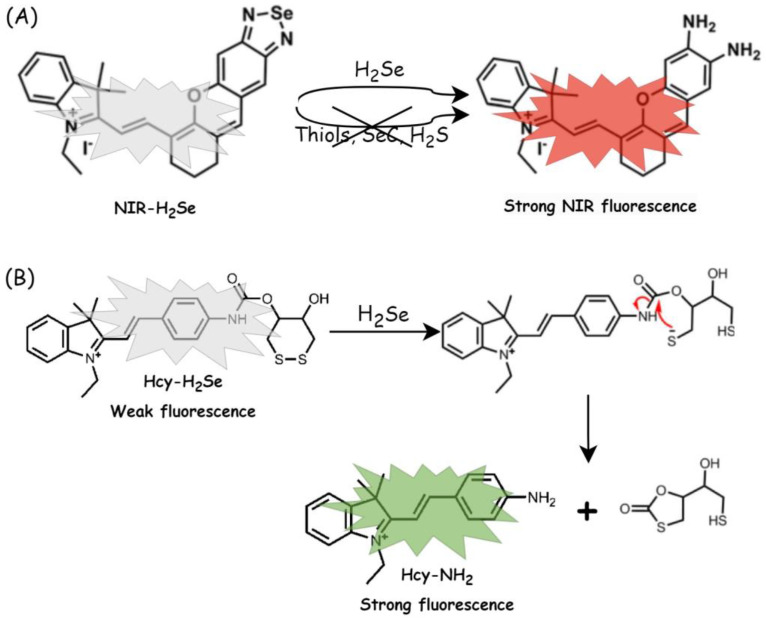
Proposed mechanism for the selective reaction of H_2_Se with fluorescent/fluorogenic probes and fluorescence turn-on of (**A**) NIR-H_2_Se ex/em = 633 nm/650–750 nm (modified from ref. [75]) and (**B**) Hcy-H_2_Se ex/em = 470 nm/575 nm (modified from ref. [76]).

In 2016, a specific fluorescent probe (NIR-H_2_Se; ex/em = 633/650–750 nm) was developed for real-time monitoring of H_2_Se in living cells and in vivo [75] (Figure 4A). Thus, it was possible to track H_2_Se in cells, and H_2_Se was shown to play a key role in cell death induced by selenite (IC_50_ = 5 μM after 24 h under normoxia). NIR-H_2_Se (10 µM) was used to detect the H_2_Se content in HepG2 cells (human hepatocellular carcinoma) during Na_2_SeO_3_-induced apoptosis under normoxic conditions via the mechanism of oxidative stress. However, in the presence of reduced oxygen, increased H_2_Se content and decreased ROS levels were observed, suggesting that cell death in a hypoxic environment occurs via a non-oxidative stress mechanism [75]. 

The results obtained with the NIR-H_2_Se probe were confirmed with another fluorescent probe, Hcy-H_2_Se (ex/em = 470 nm/575 nm, Figure 4B) [76]. The compound (10 µM), which also showed high selectivity over H_2_S, was successfully applied to image H_2_Se generated from Na_2_SeO_3_ in living cells HepG2 under both normoxic and hypoxic conditions and in solid tumors. The confocal fluorescence imaging results showed that H_2_Se gradually accumulated in a hypoxic environment, suggesting that the anticancer mechanism of Se in hypoxic solid tumors occurs via nonoxidative stress.

Later, a mitochondria-targeted fluorescent H_2_Se nanosensor, Mito-N-D-MSN, formulated from mesoporous silica nanoparticles (MSNs), was developed [90]. Mito-N-D-MSN was prepared through loading NIR-H_2_Se into MSN and modifying it with triphenylphosphonium ions (TPP) as a mitochondria-targeting moiety. The results showed that the mitochondrial H_2_Se content gradually increased after Na_2_SeO_3_ administration (at concentrations 2–10 µM and 12 h) to HepG2 cells, while O_2_ content remained unchanged in HepG2 cells under hypoxic conditions, suggesting that the anticancer mechanism of selenite involves non-oxidative stress in the tumor microenvironment. 

Next, H_2_Se release generated by Na_2_SeO_3_ under hypoxia (detected via NIR-H_2_Se probe) was shown to induce reductive stress in HepG2 cells and activate cell autophagy through regulating the redox of HMGB1 protein [73]. H_2_Se could disrupt the disulfide bond in HMGB1 and promote its secretion, which are the characteristic effects of reductive stress. The reduced HMGB1 protein was then secreted into the extracellular space, which subsequently activated autophagy through inhibiting the Akt/mTOR pathway in cells. Moreover, autophagy played a dual role; mild autophagy inhibited apoptosis, whereas excessive autophagy led to autophagy-associated cell death.

Sodium selenite, as an H_2_Se donor, was one of the selenium compounds tested in clinical trials where the combination of sodium selenite with commonly used anti-cancer therapies was applied [91,92,93,94,95].

### 4.2. Chemically Synthesized Selenium Compounds as H_2_Se Suppliers 

Chemically synthesized organic selenium-containing compounds that exhibit antitumor activity belong to different chemical classes and are metabolized into different redox-active products. Their active antitumor metabolites, similar to the dietary selenium compounds, can be divided into two groups: generators of hydrogen selenide (H_2_Se) and methylselenol (CH_3_SeH) [73]. Both are redox active but exhibit a slightly different mechanism of action and toxicity, and their properties depend on the doses used [70]. Methylselenol precursors, such as diselenides, selenoethers, selenoesters, methylseleninic acid, 1,2-benzisoselenazole-3[2H]- one, and selenophene-based derivatives, as well as selenoamino acids and selol (contains dioxaselenolane rings and selenium at +4 oxidation stage), have been addressed in the other review [18]. Moreover, the research progress and different therapeutic applications of Se-containing compounds have been reviewed [96], and many various Se-containing compounds showed potent activities such as anticancer, antioxidant, antifibrinolytic, antiparasitic, antibacterial, antiviral, antifungal, and central-nervous-system-related effects.

The toxicity and complex role of H_2_Se in human cancers requires the development of compounds that are well-defined H_2_Se donors with controllable release properties. Inorganic selenide salts (e.g., Na_2_Se and NaHSe) are short-living H_2_Se donors that not only can be toxic [97,98] but also fail to mimic slow and well-regulated H_2_Se formation in vivo. Organic Se compounds as potential H_2_Se donors are generally considered safer (lower toxicity) than inorganic Se salts [20,81,99,100,101,102,103,104]. For example, organic diselenides and phthalic selenoanhydride have shown promising biological activities, including radioprotection, free radical scavenging, and multidrug-resistance-reversing activity, but H_2_Se release was not directly observed in experiments with these compounds [81,99,100,101,102,103,104].

There were not many well-characterized and controllable H_2_Se donors that would act under physiological conditions, and the study of H_2_Se biology encountered technical difficulties because there was no reliable assay for direct H_2_Se quantification. In this review, we focus on compounds whose mechanism of action is based on hydrogen selenide, and we show how this product is formed in cases where its presence is well documented.

Due to the chemical similarity of H_2_S and H_2_Se, many researchers have been inspired by H_2_S donors in the development of synthetic H_2_Se donors.

**Figure 5:**
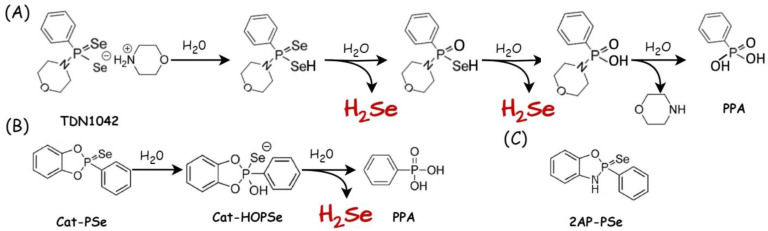
Donors for hydrolysis-based H_2_Se release. (**A**) Proposed hydrolysis mechanism of TDN1042 resulting in H_2_Se release at acidic pH (modified from ref. [106]); (**B**) hydrolysis of cyclic compound Cat-PSe to release H_2_Se at pH 7.4 (modified from [107]); (**C**) structure of 2AP-PSe used in the study [107].

The first well-characterized synthetic donor for hydrolysis-based H_2_Se release was prepared through modifying the widely used H_2_S donor GYY4137 [105]. In 2019, based on the P==S motif, the Pluth group developed the analogous compound TDN1042, which contained the P==Se motif [106]. This small synthetic molecule releases H_2_Se directly and under controlled conditions (Figure 5A). Using ^31^P and ^77^Se NMR-controlled experiments, the authors demonstrated the pH dependence of H_2_Se release (an increase in the rate at more acidic pH), and finally, they confirmed H_2_Se release using an electrophilic trapping reagent (dinitrofluorobenzene). Using HPLC, the authors observed the formation of both expected products: di(2,4-dinitrophenyl) selenide and the corresponding diselenide in the trapping solution.

Due to the relatively slow H_2_Se release at pH 7.4, the team decided to modify this compound, and in further study, they incorporated *ortho*-substituted phenols into the phosphorus center of TDN1042 to form cyclic-PSe compounds (e.g., 2AP-PSe, 2-aminophenol derivative; Cat-PSe) as H_2_Se donors (Figure 5B,C), and H_2_Se release was monitored via ^31^P and ^77^Se NMR spectroscopy using alkylative trapping experiments as before [107]. The mechanism of H_2_Se release was shown on the example of the Cat-PSe compound reaction (Figure 5B). In addition, a colorimetric method based on the reaction of H_2_Se with NBD-Cl (4-chloro-7-nitrobenzofurazan, λmax = 350 nm) was used to generate NBD-SeH (λmax = 551 nm), a compound that can be used for colorimetric detection of free H_2_Se. Moreover, TOF-SIMS (time of flight secondary ion mass spectroscopy) was used to demonstrate that 2AP-PSe is cell permeable at 10 µM concentration and in a dose-dependent manner from 0 to 25 µM. In this assay, total intracellular Se content as a function of H_2_Se donor concentration was measured.

It was also shown that 2AP-PSe can exert antioxidant activities through reducing ROS and H_2_O_2_ content in living HeLa cells.

**Figure 6:**
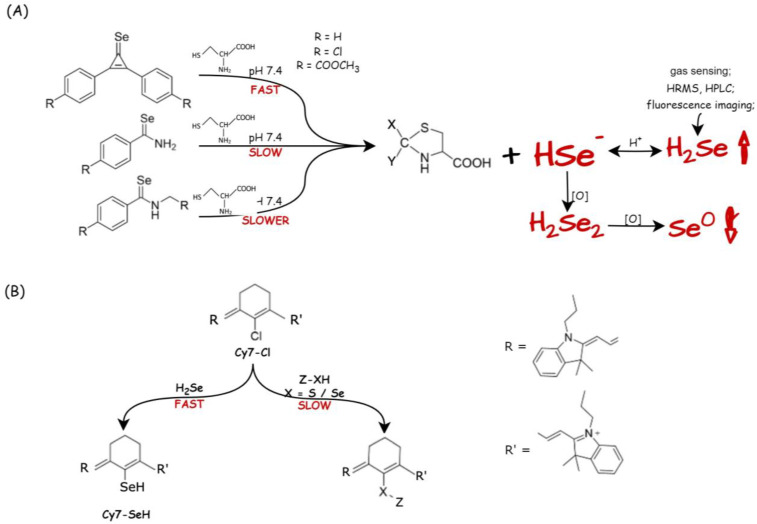
Cys-activated H_2_Se donors (modified from ref. [109]). (**A**) Comparison of H_2_Se release from selenocyclopropenone and selenoamides activated with Cys at pH 7.4. H_2_Se can be further oxidized or trapped for detection. (**B**) Quantitative detection of H_2_Se: reactions of Cy7-CI and different nucleophiles (H_2_Se, R-SeH or R-SH).

Considering the study of Pluth et al. and inspired by other synthetic H_2_S donors (compounds activated by bio-thiols [108]), Kang and coworkers proposed Cys-activated H_2_Se donors, that is, selenocarbonyl-containing derivatives with C==Se motif [109]. Thus, the C==Se motifs in selenocyclopropenones and selenoamides can be selectively activated by the -SH moiety of Cys for controllable H_2_Se release at pH 7.4, and these reactions occur with different rates for those compounds (Figure 6A). In the qualitative assay, H_2_Se release from selenocyclopropenone was observed via the formation of Se^0^ from the oxidation of H_2_Se and using the H_2_Se-selective gas detector. In addition, an electrophilic iodoacetamide was used to trap H_2_Se in this Cys-activated system, and the expected products (diacetamide selenide and diselenide) were observed via HRMS (high-resolution mass spectrometry) analysis. For the quantitative assay in aqueous solution, Cy7-CI (11-chloro-1,1′-din-propyl-3,3,3′,3′-tetramethyl-10,12-trimethyleneindatricarbocyanine iodide) was used for direct H_2_Se trapping. The selectivity of Cy7-CI was evaluated towards H_2_Se, R-SH, and R-Se, and the reaction rates were measured via HPLC and evaluated from the optical response (Figure 6B); that is, the initial absorbance peak of Cy7-CI at 780 nm was shifted to 710 nm after the reaction with H_2_Se. Cys-triggered (1 mM) H_2_Se release from arylselenoamides (25 μM) in living cells was also confirmed via confocal fluorescence imaging in HeLa cells (human cervical cancer) using the previously reported fluorescent probe NIR-H_2_Se (Figure 4, [75]). In addition, mechanistic studies of Cys-triggered H_2_Se release from both selenocyclopropenone and arylselenoamide were performed.

**Figure 7:**
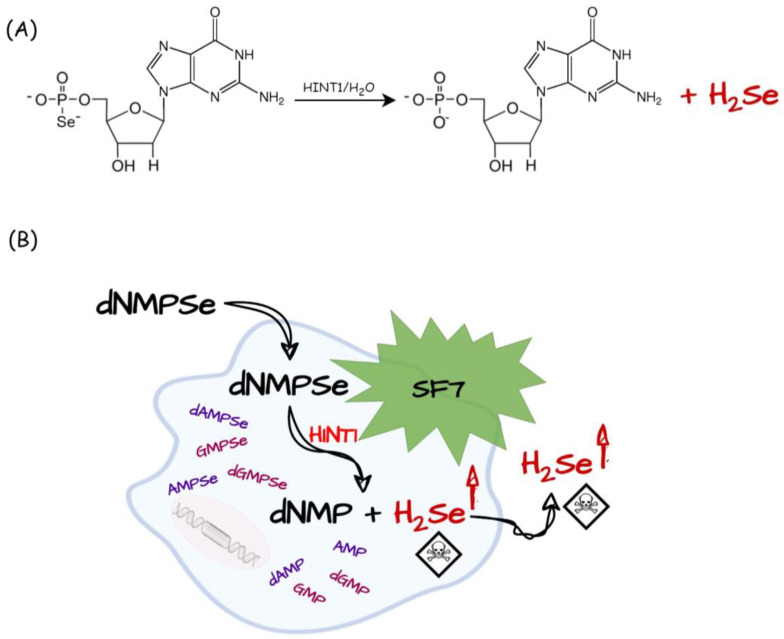
(**A**) Hydrolysis of dGMPSe catalyzed by HINT1 with the release of H_2_Se; (**B**) the release of H_2_Se in cells as a consequence of HINT1-assisted conversion of (d)NMPSe to (d)NMP (modified from ref. [110]).

Recently, selenium-derived nucleotides have been proposed as a new class of selenium-containing compounds that are H_2_Se donors with anticancer potential. These compounds have been shown to act through a novel mechanism of H_2_Se release in cells, and the involvement of the HINT1 protein in this process has been demonstrated [110]. HINT1 is a hydrolase involved in nucleoside/nucleotide metabolism. This cytoplasmic enzyme is widely expressed in all organisms and belongs to the histidine triad superfamily (HIT), whose members are characterized by the sequence motif H-X-H-X-H-X-X-X (H—a histidine residue, X—a hydrophobic amino acid) [111]. Previously, HINT1 was shown to be involved in the metabolism of phosphorothioate oligonucleotide drugs catalyzing the conversion of 5′-O-thiophosphate nucleosides (NMPS) to NMP and H_2_S in vitro and in cells [112,113]. By analogy, studies have shown that deoxyguanosine selenophosphate (dGMPSe) undergoes HINT1-assisted hydrolysis to the corresponding phosphate and H_2_Se in a similar manner both in vitro and intracellularly, according to the equation presented in Figure 7A.

The release of H_2_Se in the living cells and the tube after administration of dGMPSe was observed using a fluorogenic SF7 probe (5 μM, ex/em = 485/528 nm, Figure 7B), which has been used previously to detect the release of H_2_S in cells [113,114]. Moreover, the toxicity of dGMPSe to HeLa cells was determined (IC_50_ = 8 µM after 24 h), and the induction of tumor cell death has been shown to be related to H_2_Se release, which was observed after only 6 h of incubation in cells at dGMPSe concentration of 10 µM. In contrast to dGMPSe, dGMPS was not cytotoxic to HeLa cells even at millimolar concentrations. Because of the substrate specificity of HINT1, the reaction with the enzyme can be extended to other nucleoside 5′-*O*-selenophosphates {(d)NMPSe}. Consistent with the activity shown for various nucleoside phosphorothioates of the ribo- and deoxy- series (higher activity for purine ribonucleosides), the cellular toxic effect toward AMPSe and GMPSe may also be greater than for dGMPSe [115].

**Figure 8:**
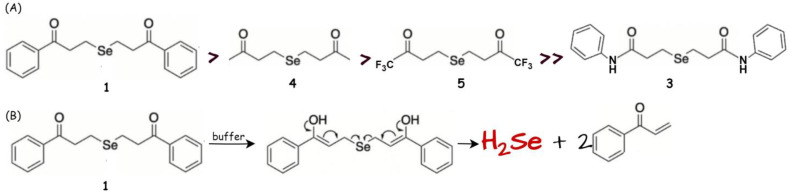
(**A**) Library of γ-keto selenides donors arranged in order of increasing inhibition of cell growth (no. 1 is the best inhibitor) (modified from ref. [116]). (**B**) Enol-mediated release of H_2_Se from γ-keto selenides. H_2_Se donor promoted by a base.

Recently, Lukesh and coworkers reported that γ-keto selenides provide a useful platform for H_2_Se donation via an α-deprotonation/β-elimination pathway that is highly dependent on both pH and alpha-proton acidity [116]. This new class of slow-release donors functioned via an alternative mechanism compared to the first compound reported by the Pluth team [106], and the rate of H_2_Se release was enhanced under neutral to slightly basic conditions (Figure 8B), in contrast to the increased hydrolysis at more acidic pH for TDN1042. To unequivocally confirm H_2_Se release from γ-keto selenides, an additional trapping experiment was performed. Using HRMS, the resulting product, volatilized H_2_Se, was captured with electrophilic iodoacetamide and easily detected in the obtained spectrum. Further evidence for the release of H_2_Se from γ-keto selenides was the formation of more stable selenodiglutathione (GSSeSG) upon its reaction in a redox buffer consisting of glutathione and glutathione disulfide in an ammonium bicarbonate buffer (observed directly via mass spectrometry). In a cellular environment, the γ-keto selenides compounds were evaluated for their growth inhibitory activity against HeLa and HCT116 cells (human colon cancer). The investigated H_2_Se donors 1, 4, 5 (Figure 8A) showed low micromolar activity against both studied cancer cell lines (IC_50_ = 3.7–10.6 µM in HeLa; IC_50_ = 4.1–10.1 µM in HCT116 and >50 µM in the case of control, compound 3 for both cell lines), which is consistent with the anticancer activity of the previously reported H_2_Se donors [74,110].

The selenium compounds discussed in this review are presented in Table 1.

## 5. Conclusions

Many pathological conditions, including cancer, may be due to an imbalance of oxidative and reductive byproducts. To protect their cell populations from damage, organisms have conserved stress response pathways that recognize and mitigate a variety of adverse conditions. Because of their rapid activation, stress responses should be terminated soon after cellular homeostasis is restored. Otherwise, the cell is threatened with dire consequences, even death.

Reductive stress is not as well-characterized of a phenomenon as oxidative stress. It has only recently gained more attention, particularly because of its potential importance and therapeutic intervention in cancer. Understanding the regulation of responses to reductive stress both in normal cells and in the tumor microenvironment can provide new therapeutic approaches for cancer treatment and anticancer drug resistance. Therefore, compounds that can selectively and controllably induce reductive stress in cells are being sought. Selenium-containing compounds which are H_2_Se donors meet these requirements.

However, there are few detailed studies on the chemical biology of H_2_Se, and there are not many reports about the essential physiological functions of H_2_Se, the cellular objects, and the therapeutic perspectives. The lack of clarity on these key questions was largely due to the lack of small molecule donors that could effectively enhance the bioavailability of H_2_Se through continuously releasing the unstable biomolecule under physiologically relevant conditions.

In this review, we present only H_2_Se donors where the release of this product has been documented using various techniques such as fluorescence imaging, HRMS, ^77^SeNMR, or others. Previously (before 2016), there were some compounds for which H_2_Se release was supposed but for which there was no technical evidence. Since then, several H_2_Se donors have been developed for which H_2_Se release has been well documented, although not all of them have been demonstrated to show anticancer activity in the cellular model. However, the possibility of using several new H_2_Se donors provides an opportunity to explain their influence on redox homeostasis of cancer cells and to answer the question of whether Se is an antioxidant or a pro-oxidant. So far, it has been shown that depending on aerobic conditions, selenium compounds can act as generators of oxidative stress (normoxia) or reductive stress (hypoxia). However, it is too early to compare their efficacy in cancer therapy with other selenium-containing compounds as well the mechanisms responsible for their toxicity in cells as only Na_2_SeO_3_ was studied so deeply (apoptosis under oxidative stress and autophagy under reductive stress). Many other mechanisms are possible, including different types cell death such as ferroptosis, necroptosis, etc.; influence on DNA repair or damage; and other various cell signaling pathways.

In conclusion, future studies are needed to show the utility of selenium compounds as H_2_Se donors in cancer therapy and in the selective induction of reductive stress in cells and in vivo, as few data are available to date.

## Figures and Tables

**Table 1:** Selenium compounds as H_2_Se donors and studies of their biological effects.

Selenium Compound	Detection Method of H_2_Se Release	Mechanism of H_2_Se Release	Biological Model	Ref.
Na_2_SeO_3_ (under clinical trials)	Fluorescence imaging NIR-H_2_Se	Enzymatic: Grx (GSH), Trx, TrxR (NADPH)	HepG2 cells (cytotoxicity, reductive stress, H_2_Se release), mice	[74,75]
	Fluorescence imaging Hcy-H_2_Se		HepG2 (H_2_Se release)	[76]
	Fluorescence imaging Mito-N-D-MSN (nanoprobes mitochondria-targeted)		HepG2 (H_2_Se release)	[90]
TDN1042 (P=Se motif)	^31^P and ^77^Se NMR and electrophilic trapping reagent	Acidic conditions	-	[106]
2AP-PSe, Cat-PSe (P=Se motif)	^31^P and ^77^Se NMR and electrophilic trapping reagent; colorimetric detection with NBD-Cl	pH 7.2	HeLa cells (antioxidant activity)	[107]
selenocyclopropenones and arylselenoamides (C=Se motif)	H_2_Se-selective gas detector; electrophilic trapping reagent and HRMS analysis; Cy7-CI trapping; fluorescence imaging (NIR-H_2_Se)	Cys at pH 7.4	HeLa cells (Cys-mediated H_2_Se release)	[109]
dGMPSe	Fluorescence imaging SF7	Enzymatic: HINT1	HeLa cells (cytotoxicity and H_2_Se release)	[110]
γ-keto selenides	Trapping reagent and HRMS	neutral to slightly basic conditions	HeLa and HCT116 cells (cytotoxicity)	[116]

## Data Availability

Not applicable.

## References

[1] Quiles J.L., Sánchez-González C., Vera-Ramírez L., Giampieri F., Navarro-Hortal M.D., Xiao J., Llopis J., Battino M., Varela-López A. (2020). Reductive Stress, Bioactive Compounds, Redox-Active Metals, and Dormant Tumor Cell Biology to Develop Redox-Based Tools for the Treatment of Cancer. Antioxid. Redox Signal..

[2] Sabharwal S.S., Schumacker P.T. (2014). Mitochondrial ROS in cancer: Initiators, amplifiers or an Achilles’ heel?. Nat. Rev. Cancer.

[3] Xiao W., Loscalzo J. (2020). Metabolic Responses to Reductive Stress. Antioxid. Redox Signal..

[4] Yang Y., Sauve A.A. (2016). NAD+ metabolism: Bioenergetics, signaling and manipulation for therapy. Biochim. Biophys. Acta.

[5] Sarsour E.H., Kumar M.G., Chaudhuri L., Kalen A.L., Goswami P.C., Liu G.-Y., Sun Y.-Z., Zhou N., Du X.-M., Yang J. (2009). Redox Control of the Cell Cycle in Health and Disease. Antioxid. Redox Signal..

[6] Handy D., Loscalzo J. (2012). Redox Regulation of Mitochondrial Function. Antioxid. Redox Signal..

[7] Handy D.E., Loscalzo J. (2017). Responses to reductive stress in the cardiovascular system. Free. Radic. Biol. Med..

[8] Korge P., Calmettes G., Weiss J.N. (2015). Increased reactive oxygen species production during reductive stress: The roles of mitochondrial glutathione and thioredoxin reductases. Biochim. Biophys. Acta.

[9] Chun K.-S., Kim D.-H., Surh Y.-J. (2021). Role of Reductive versus Oxidative Stress in Tumor Progression and Anticancer Drug Resistance. Cells.

[10] Hayes J.D., Dinkova-Kostova A.T., Tew K.D. (2020). Oxidative Stress in Cancer. Cancer Cell.

[11] George S., Abrahamse H. (2020). Redox Potential of Antioxidants in Cancer Progression and Prevention. Antioxidants.

[12] Ma W.-X., Li C.-Y., Tao R., Wang X.-P., Yan L.-J. (2020). Reductive Stress-Induced Mitochondrial Dysfunction and Cardiomyopathy. Oxidative Med. Cell. Longev..

[13] Tretter V., Hochreiter B., Zach M.L., Krenn K., Klein K.U. (2021). Understanding Cellular Redox Homeostasis: A Challenge for Precision Medicine. Int. J. Mol. Sci..

[14] Zou Z., Chang H., Li H., Wang S. (2017). Induction of Reactive Oxygen Species: An Emerging Approach for Cancer Therapy. Apoptosis.

[15] Zhang L., Tew K.D. (2021). Chapter Ten-Reductive stress in cancer. Adv. Cancer Res..

[16] Rankin E.B., Giaccia A.J. (2016). Hypoxic control of metastasis. Science.

[17] Fernandes A.P., Gandin V. (2015). Selenium compounds as therapeutic agents in cancer. Biochim. Biophys. Acta BBA-Gen. Subj..

[18] Radomska D., Czarnomysy D., Radomski D., Bielawski K. (2021). Selenium Compounds as Novel Potential Anticancer Agents. Int. J. Mol. Sci..

[19] Kim S.J., Choi M.C., Park J.M., Chung A.S. (2021). Antitumor Effects of Selenium. Int. J. Mol. Sci..

[20] Weekley C.M., Harris H.H. (2013). Which form is that? The importance of selenium speciation and metabolism in the prevention and treatment of disease. Chem. Soc. Rev..

[21] Gores G.J., Flarsheim C.E., Dawson T.L., Nieminen A.L., Herman B., Lemasters J.J. (1989). Swelling, reductive stress, and cell death during chemical hypoxia in hepatocytes. Am. J. Physiol. Physiol..

[22] Paniker N., Srivastava S., Beutler E. (1970). Glutathione metabolism of the red cells effect of glutathione reductase deficiency on the stimulation of hexose monophosphate shunt under oxidative stress. Biochim. Biophys. Acta BBA-Gen. Subj..

[23] Holmström K.M., Finkel T. (2014). Cellular mechanisms and physiological consequences of redox-dependent signalling. Nat. Rev. Mol. Cell Biol..

[24] Buettner G.R. (2011). Superoxide Dismutase in Redox Biology: The Roles of Superoxide and Hydrogen Peroxide. Anti-Cancer Agents Med. Chem..

[25] Itoh K., Chiba T., Takahashi S., Ishii T., Igarashi K., Katoh Y., Oyake T., Hayashi N., Satoh K., Hatayama I. (1997). An Nrf2/small Maf heterodimer mediates the induction of phase II detoxifying enzyme genes through antioxidant response elements. Biochem. Biophys. Res. Commun..

[26] Rajasekaran N.S., Connell P., Christians E.S., Yan L.-J., Taylor R.P., Orosz A., Zhang X.Q., Stevenson T.J., Peshock R.M., Leopold J.A. (2007). Human αB-Crystallin Mutation Causes Oxido-Reductive Stress and Protein Aggregation Cardiomyopathy in Mice. Cell.

[27] Yamamoto M., Kensler T.W., Motohashi H. (2018). The KEAP1-NRF2 System: A Thiol-Based Sensor-Effector Apparatus for Maintaining Redox Homeostasis. Physiol. Rev..

[28] Ishii T., Itoh K., Takahashi S., Sato H., Yanagawa T., Katoh Y., Bannai S., Yamamoto M. (2000). Transcription Factor Nrf2 Coordinately Regulates a Group of Oxidative Stress-inducible Genes in Macrophages. J. Biol. Chem..

[29] Kobayashi E.H., Suzuki T., Funayama R., Nagashima T., Hayashi M., Sekine H., Tanaka N., Moriguchi T., Motohashi H., Nakayama K. (2016). Nrf2 suppresses macrophage inflammatory response by blocking proinflammatory cytokine transcription. Nat. Commun..

[30] Suzuki T., Hidaka T., Kumagai Y., Yamamoto M. (2020). Environmental pollutants and the immune response. Nat. Immunol..

[31] Rodríguez-Colman M.J., Schewe M., Meerlo M., Stigter E., Gerrits J., Pras-Raves M., Sacchetti A., Hornsveld M., Oost K.C., Snippert H.J. (2017). Interplay between metabolic identities in the intestinal crypt supports stem cell function. Nature.

[32] Sena L.A., Chandel N.S. (2012). Physiological roles of mitochondrial reactive oxygen species. Mol. Cell.

[33] Padmanabhan B., Tong K.I., Ohta T., Nakamura Y., Scharlock M., Ohtsuji M., Kang M.-I., Kobayashi A., Yokoyama S., Yamamoto M. (2006). Structural Basis for Defects of Keap1 Activity Provoked by Its Point Mutations in Lung Cancer. Mol. Cell.

[34] Shibata T., Ohta T., Tong K.I., Kokubu A., Odogawa R., Tsuta K., Asamura H., Yamamoto M., Hirohashi S. (2008). Cancer related mutations in *NRF2* impair its recognition by Keap1-Cul3 E3 ligase and promote malignancy. Proc. Natl. Acad. Sci. USA.

[35] Romero R., Sayin V.I., Davidson S.M., Bauer M.R., Singh S.X., Leboeuf S.E., Karakousi T.R., Ellis D.C., Bhutkar A., Sánchez-Rivera F.J. (2017). Keap1 loss promotes Kras-driven lung cancer and results in dependence on glutaminolysis. Nat. Med..

[36] Buckley S.M., Aranda-Orgilles B., Strikoudis A., Apostolou E., Loizou E., Moran-Crusio K., Farnsworth C.L., Koller A.A., Dasgupta R., Silva J.C. (2012). Regulation of Pluripotency and Cellular Reprogramming by the Ubiquitin-Proteasome System. Cell Stem Cell.

[37] Akopian D., Rape M. (2018). Principles of Ubiquitin-Dependent Signaling. Annu. Rev. Cell Dev. Biol..

[38] Yau R., Rape M. (2016). The increasing complexity of the ubiquitin code. Nature.

[39] Rape M. (2017). Ubiquitylation at the crossroads of development and disease. Nat. Rev. Mol. Cell Biol..

[40] Zhang D.D., Lo S.-C., Cross J.V., Templeton D.J., Hannink M. (2004). Keap1 Is a Redox-Regulated Substrate Adaptor Protein for a Cul3-Dependent Ubiquitin Ligase Complex. Mol. Cell. Biol..

[41] Bellezza I., Giambanco I., Minelli A., Donato R. (2018). Nrf2-Keap1 signaling in oxidative and reductive stress. Biochim. Biophys. Acta BBA-Mol. Cell Res..

[42] Tong K.I., Katoh Y., Kusunoki H., Itoh K., Tanaka T., Yamamoto M. (2006). Keap1 Recruits Neh2 through Binding to ETGE and DLG Motifs: Characterization of the Two-Site Molecular Recognition Model. Mol. Cell. Biol..

[43] Eggler A.L., Liu G., Pezzuto J.M., Van Breemen R.B., Mesecar A.D. (2005). Modifying specific cysteines of the electrophile-sensing human Keap1 protein is insufficient to disrupt binding to the Nrf2 domain Neh2. Proc. Natl. Acad. Sci. USA.

[44] Kobayashi M., Li L., Iwamoto N., Nakajima-Takagi Y., Kaneko H., Nakayama Y., Eguchi M., Wada Y., Kumagai Y., Yamamoto M. (2009). The Antioxidant Defense System Keap1-Nrf2 Comprises a Multiple Sensing Mechanism for Responding to a Wide Range of Chemical Compounds. Mol. Cell. Biol..

[45] Suzuki T., Muramatsu A., Saito R., Iso T., Shibata T., Kuwata K., Kawaguchi S.-I., Iwawaki T., Adachi S., Suda H. (2019). Molecular Mechanism of Cellular Oxidative Stress Sensing by Keap1. Cell Rep..

[46] Furukawa M., Xiong Y. (2005). BTB Protein Keap1 Targets Antioxidant Transcription Factor Nrf2 for Ubiquitination by the Cullin 3-Roc1 Ligase. Mol. Cell. Biol..

[47] Kaelin W.G. (2007). Von Hippel-Lindau disease. Annu. Rev. Pathol..

[48] Lu H., Samanta D., Xiang L., Zhang H., Hu H., Chen I., Bullen J.W., Semenza G.L. (2015). Chemotherapy triggers HIF-1–dependent glutathione synthesis and copper chelation that induces the breast cancer stem cell phenotype. Proc. Natl. Acad. Sci. USA.

[49] Wakabayashi N., Itoh K., Wakabayashi J., Motohashi H., Noda S., Takahashi S., Imakado S., Kotsuji T., Otsuka F., Roop D.R. (2003). Keap1-null mutation leads to postnatal lethality due to constitutive Nrf2 activation. Nat. Genet..

[50] Gnarra J.R., Ward J.M., Porter F.D., Wagner J.R., Devor D.E., Grinberg A., Emmert-Buck M.R., Westphal H., Klausner R.D., Linehan W.M. (1997). Defective placental vasculogenesis causes embryonic lethality in VHL-deficient mice. Proc. Natl. Acad. Sci. USA.

[51] Cancer Genome Atlas Research Network (2012). Comprehensive genomic characterization of squamous cell lung cancers. Nature.

[52] Velichkova M., Hasson T. (2005). Keap1 regulates the oxidation-sensitive shuttling of Nrf2 into and out of the nucleus via a Crm1-dependent nuclear export mechanism. Mol. Cell. Biol..

[53] Bellezza I., Mierla A.L., Minelli A. (2010). Nrf2 and NF-κB and their concerted modulation in cancer pathogenesis and progression. Cancer.

[54] Liu Y., Li Q., Zhou L., Xie N., Nice E.C., Zhang H., Huang C., Lei Y. (2016). Cancer drug resistance: Redox resetting renders a way. Oncotarget.

[55] McClung J.P., Roneker C.A., Mu W., Lisk D.J., Langlais P., Liu F., Lei X.G. (2004). Development of insulin resistance and obesity in mice overexpressing cellular glutathione peroxidase. Proc. Natl. Acad. Sci. USA.

[56] Dialynas G., Shrestha O.K., Ponce J.M., Zwerger M., Thiemann D.A., Young G.H., Moore S.A., Yu L., Lammerding J., Wallrath L.L. (2015). Myopathic lamin mutations cause reductive stress and activate the Nrf2/Keap-1 pathway. PLoS Genet..

[57] Bjelakovic G., Nikolova D., Gluud L.L., Simonetti R.G., Gluud C. (2007). Mortality in randomized trials of antioxidant supplements for primary and secondary prevention: Systematic review and meta-analysis. JAMA.

[58] Manford A.G., Rodríguez-Pérez F., Shih K.Y., Shi Z., Berdan C.A., Choe M., Titov D.V., Nomura D.K., Rape M. (2020). A cellular mechanism to detect and alleviate reductive stress. Cell.

[59] Manford A.G., Mena E.L., Shih K.Y., Gee C.L., McMinimy R., Martínez-González B., Sherriff R., Lew B., Zoltek M., Rodríguez-Pérez F. (2021). Structural basis and regulation of the reductive stress response. Cell.

[60] Henning N.J., Manford A.G., Spradlin J.N., Brittain S.M., Zhang E., McKenna J.M., Tallarico J.A., Schirle M., Rape M., Nomura D.K. (2022). Discovery of a Covalent FEM1B Recruiter for Targeted Protein Degradation Applications. J. Am. Chem. Soc..

[61] Gao X., Wei K., Hu B., Xu K., Tang B. (2019). Ascorbic acid induced HepG2 cells’ apoptosis via intracellular reductive stress. Theranostics.

[62] Kipp A.P., Frombach J., Deubel S., Brigelius-Flohé R. (2013). Selenoprotein W as Biomarker for the Efficacy of Selenium Compounds to Act as Source for Selenoprotein Biosynthesis. Methods Enzymol..

[63] Ganyc D., Self W.T. (2007). High affinity selenium uptake in a keratinocyte model. FEBS Lett..

[64] Mizutani T., Kurata H., Yamada K. (1991). Study of mammalian selenocysteyl-tRNA synthesis with [^75^Se]HSe^−^. FEBS Lett..

[65] Turanov A.A., Xu X.-M., Carlson B.A., Yoo M.-H., Gladyshev V.N., Hatfield D.L. (2011). Biosynthesis of Selenocysteine, the 21st Amino Acid in the Genetic Code, and a Novel Pathway for Cysteine Biosynthesis. Adv. Nutr. Int. Rev. J..

[66] Combs G.F. (2015). Biomarkers of Selenium Status. Nutrients.

[67] Sierant M., Leszczynska G., Sadowska K., Komar P., Radzikowska-Cieciura E., Sochacka E., Nawrot B. (2018). *Escherichia coli* tRNA 2-selenouridine synthase (SelU) converts S2U-RNA to Se2U-RNA via S-geranylated-intermediate. FEBS Lett..

[68] Szczupak P., Sierant M., Wielgus E., Radzikowska-Cieciura E., Kulik K., Krakowiak A., Kuwerska P., Leszczynska G., Nawrot B. (2022). *Escherichia coli* tRNA 2-Selenouridine Synthase (SelU): Elucidation of Substrate Specificity to Understand the Role of *S*-Geranyl-tRNA in the Conversion of 2-Thio- into 2-Selenouridines in Bacterial tRNA. Cells.

[69] Sun H., Sheng J., Hassan A.E., Jiang S., Gan J., Huang Z. (2012). Novel RNA base pair with higher specificity using single seleniumatom. Nucleic Acids Res..

[70] Sanmartín C., Plano D., Sharma A.K., Palop J.A. (2012). Selenium compounds, apoptosis and other types of cell death: An overview for cancer therapy. Int. J. Mol. Sci..

[71] Ip C., Thompson H.J., Zhu Z., Ganther H.E. (2000). In vitro and in vivo studies of methylseleninic acid: Evidence that a monomethylated selenium metabolite is critical for cancer chemoprevention. Cancer Res..

[72] Lu J., Jiang C. (2005). Selenium and Cancer Chemoprevention: Hypotheses Integrating the Actions of Selenoproteins and Selenium Metabolites in Epithelial and Non-Epithelial Target Cells. Antioxid. Redox Signal..

[73] Varlamova E.G., Turovsky E.A. (2021). The main cytotoxic effects of methylseleninic acid on various cancer cells. Int. J. Mol. Sci..

[74] Pan X., Song X., Wang C., Cheng T., Luan D., Xu K., Tang B. (2019). H_2_Se Induces Reductive Stress in HepG2 Cells and Activates Cell Autophagy by Regulating the Redox of HMGB1 Protein under Hypoxia. Theranostics.

[75] Kong F., Ge L., Pan X., Xu K., Liu X., Tang B. (2015). A highly selective near-infrared fluorescent probe for imaging H_2_Se in living cells and in vivo. Chem. Sci..

[76] Kong F., Zhao Y., Liang Z., Liu X., Pan X., Luan D., Xu K., Tang B. (2017). Highly Selective Fluorescent Probe for Imaging H_2_Se in Living Cells and in Vivo Based on the Disulfide Bond. Anal. Chem..

[77] Nuttall K.L., Allen F.S. (1984). Kinetics of the reaction between hydrogen selenide ion and oxygen. Inorg. Chim. Acta.

[78] Cupp-Sutton K.A., Ashby M.T. (2016). Biological Chemistry of Hydrogen Selenide. Antioxidants.

[79] Cao W., Li X., Zheng S., Zheng W., Wong Y.-S., Chen T. (2014). Selenocysteine derivative overcomes TRAIL resistance in melanoma cells: Evidence for ROS-dependent synergism and signaling crosstalk. Oncotarget.

[80] Zhang K., Su J., Chen D., Lin B., Wu Y., Wang Y., Lei J., Zheng R., Zhu B., Li Y. (2022). L-Selenocysteine induced HepG-2 cells apoptosis through reactive oxygen species-mediated signaling pathway. Mol. Biol. Rep..

[81] Gandin V., Khalkar P., Braude J., Fernandes A.P. (2018). Organic selenium compounds as potential chemotherapeutic agents for improved cancer treatment. Free. Radic. Biol. Med..

[82] Weekley C., Aitken J.B., Musgrave I., Harris H.H. (2012). Methylselenocysteine Treatment Leads to Diselenide Formation in Human Cancer Cells: Evidence from X-ray Absorption Spectroscopy Studies. Biochemistry.

[83] Weekley C.M., Aitken J.B., Vogt S., Finney L.A., Paterson D.J., de Jonge M.D., Howard D.L., Musgrave I.F., Harris H.H. (2011). Uptake, Distribution, and Speciation of Selenoamino Acids by Human Cancer Cells: X-ray Absorption and Fluorescence Methods. Biochemistry.

[84] Roman M., Jitaru P., Barbante C. (2013). Selenium biochemistry and its role for human health. Metallomics.

[85] Kim E., Sohn S., Kwon H., Kim S., Kim M. (2007). Sodium selenite induces superoxide-mediated mitochondrial damage and subsequent autophagic cell death in malignant glioma cells. Cancer Res..

[86] Xiang N., Zhao R., Zhong W. (2009). Sodium selenite induces apoptosis by generation of superoxide via the mitochondrial-dependent pathway in human prostate cancer cells. Cancer Chemother. Pharmacol..

[87] Olm E., Fernandes A.P., Hebert C., Rundlöf A.-K., Larsen E.H., Danielsson O., Björnstedt M. (2009). Extracellular thiol-assisted selenium uptake dependent on the xc− cystine transporter explains the cancer-specific cytotoxicity of selenite. Proc. Natl. Acad. Sci. USA.

[88] Weekley C.M., Aitken J.B., Vogt S., Finney L.A., Paterson D.J., de Jonge M.D., Howard D.L., Witting P.K., Musgrave I.F., Harris H.H. (2011). Metabolism of Selenite in Human Lung Cancer Cells: X-Ray Absorption and Fluorescence Studies. J. Am. Chem. Soc..

[89] Chen P., Wang L., Li N., Liu Q., Ni J. (2013). Comparative proteomics analysis of sodium selenite induced apoptosis in human prostate cancer cells. Metallomics.

[90] Cheng R., Kong F., Tong L., Liu X., Xu K., Tang B. (2018). Simultaneous Detection of Mitochondrial Hydrogen Selenide and Superoxide Anion in HepG2 Cells under Hypoxic Conditions. Anal. Chem..

[91] ClinicalTrials.gov High Dose Inorganic Selenium for Preventing Chemotherapy Induced Peripheral Neuropathy (SELENIUM). Phase 3, Recruiting. https://clinicaltrials.gov/ct2/show/NCT04201561.

[92] ClinicalTrials.gov Sodium Selenite and Radiation Therapy in Treating Patients with Metastatic Cancer. Phase 1, Completed. https://clinicaltrials.gov/ct2/show/NCT02184533.

[93] ClinicalTrials.gov The Use of Selenium to Treat Secondary Lymphedema—Breast Cancer. Phase 2, Completed. https://clinicaltrials.gov/ct2/show/NCT00188604.

[94] ClinicalTrials.gov Sodium Selenite as a Cytotoxic Agent in Advanced Carcinoma (SECAR). Phase 2, Unknown. https://clinicaltrials.gov/ct2/show/NCT01959438.

[95] ClinicalTrials.gov Phase I Sodium Selenite in Combination with Docetaxel in Castration-resistant Prostate Cancer. Phase 1, Terminated. https://clinicaltrials.gov/ct2/show/NCT01155791.

[96] Chuai H., Zhang S.-Q., Bai H., Li J., Wang Y., Sun J., Wen E., Zhang J., Xin M. (2021). Small molecule selenium-containing compounds: Recent development and therapeutic applications. Eur. J. Med. Chem..

[97] Lu J., Jiang C., Kaeck M., Ganther H., Vadhanavikit S., Clement I.P., Thompson H. (1995). Dissociation of the genotoxic and growth inhibitory effects of selenium. Biochem. Pharmacol..

[98] Peyroche G., Saveanu C., Dauplais M., Lazard M., Beuneu F., Decourty L., Malabat C., Jacquier A., Blanquet S., Plateau P. (2012). Sodium selenide toxicity is mediated by O_2_-dependent DNA breaks. PLoS ONE.

[99] Brozmanová J., Mániková D., Vlcková V., Chovanec M. (2010). Selenium: A double-edged sword for defense and offence in cancer. Arch. Toxicol..

[100] Ringuet M.T., Hunne B., Lenz M., Bravo D.M., Furness J.B. (2021). Analysis of bioavailability and induction of glutathione peroxidase by dietary nanoelemental, organic and inorganic selenium. Nutrients.

[101] Kunwar A., Mishra B., Barik A., Kumbhare L.B., Pandey R., Jain V.K., Priyadarsini K.I. (2007). 3,3′-Diselenodipropionic acid, an efficient peroxyl radical scavenger and a GPx mimic, protects erythrocytes (RBCs) from AAPH-induced hemolysis. Chem. Res. Toxicol..

[102] Domínguez-Álvarez E., Plano D., Font M., Calvo A., Prior C., Jacob C., Palop J.A., Sanmartín C. (2014). Synthesis and antiproliferative activity of novel selenoester derivatives. Eur. J. Med.Chem..

[103] Gajdács M., Spengler G., Sanmartín C., Marć M.A., Handzlik J., Domínguez-Álvarez E. (2017). Selenoesters andselenoanhydrides as novel multidrug resistance reversing agents: Aconfirmation study in a colon cancer MDR cell line. Bioorg. Med.Chem. Lett..

[104] Kharma A., Misak A., Grman M., Brezova V., Kurakova L., Baráth P., Jacob C., Chovanec M., Ondrias K., Domínguez-Álvarez E. (2019). Release of reactive seleniumspecies from phthalic selenoanhydride in the presence of hydrogensulfide and glutathione with implications for cancer research. New J. Chem..

[105] Li L., Whiteman M., Guan Y.Y., Neo K.L., Cheng Y., Lee S.W., Zhao Y., Baskar R., Tan C.H., Moore P.K. (2008). Characterization of a novel, water-soluble hydrogen sulfide-releasing molecule (GYY4137): New insights into the biology of hydrogen sulfide. Circulation.

[106] Newton T.D., Pluth M.D. (2019). Development of a hydrolysis-based small-molecule hydrogen selenide (H_2_Se) donor. Chem. Sci..

[107] Newton T.D., Bolton S.G., Garcia A.C., Chouinard J.E., Golledge S.L., Zakharov L.N., Pluth M.D. (2021). Hydrolysis-Based Small-Molecule Hydrogen Selenide (H2Se) Donors for Intracellular H2Se Delivery. J. Am. Chem. Soc..

[108] Levinn C.M., Cerda M.M., Pluth M.D. (2020). Activatable Small-Molecule Hydrogen Sulfide Donors. Antioxid. Redox Signal..

[109] Kang X., Huang H., Jiang C., Cheng L., Sang Y., Cai X., Dong Y., Sun L., Wen X., Xi Z. (2022). Cysteine-Activated Small-Molecule H2Se Donors Inspired by Synthetic H2S Donors. J. Am. Chem. Soc..

[110] Krakowiak A., Czernek L., Pichlak M., Kaczmarek R. (2022). Intracellular HINT1-Assisted Hydrolysis of Nucleoside 5′-*O*-Selenophosphate Leads to the Release of Hydrogen Selenide That Exhibits Toxic Effects in Human Cervical Cancer Cells. Int. J. Mol. Sci..

[111] Martin J., St-Pierre M.V., Dufour J.F. (2011). Hit proteins, mitochondria and cancer. Biochim. Biophys. Acta.

[112] Krakowiak A., Pawłowska R., Kocoń-Rębowska B., Dolot R., Stec W.J. (2014). Interactions of cellular histidine triad nucleotide binding protein 1 with nucleosides 5’-O-monophosphorothioate and their derivatives-Implication for desulfuration process in the cell. Biochim. Biophys. Acta.

[113] Krakowiak A., Piotrzkowska D., Kocoń-Rębowska B., Kaczmarek R., Maciaszek A. (2019). The role of the Hint1 protein in the metabolism of phosphorothioate oligonucleotides drugs and prodrugs, and the release of H2S under cellular conditions. Biochem. Pharmacol..

[114] Lin V.S., Lippert A.R., Chang C.J. (2013). Cell-trappable fluorescent probes for endogenous hydrogen sulfide signaling and imaging H_2_O_2_-dependent H2S production. Proc. Natl. Acad. Sci. USA.

[115] Ozga M., Dolot R., Janicka M., Kaczmarek R., Krakowiak A. (2010). Histidine Triad Nucleotide-binding Protein 1 (HINT-1) Phosphoramidase Transforms Nucleoside 5′-O-Phosphorothioates to Nucleoside 5′-O-Phosphates. J. Biol. Chem..

[116] Hankins R.A., Carter M.E., Zhu C., Chen C., Lukesh J.C. (2022). 3rd. Enol-mediated delivery of H2Se from γ-keto selenides: Mechanistic insight and evaluation. Chem. Sci..

